# Supplementary material for: TGFβ-dependent expression of PD-1 and PD-L1 controls CD8+ T cell anergy in transplant tolerance
Source: eLife. 2016 Jan 29;5:e08133. doi: 10.7554/eLife.08133 (PMC4749558; doi:10.7554/eLife.08133)
Supplement: Figure 8—source data 1. — DOI: http://dx.doi.org/10.7554/eLife.08133.018 [file elife-08133-fig8-data1.doc]

**Figure 8-Source data 1: Genes upregulated in anergic CD8^+^ T cells from CD3 Ab-treated recipient mice**

| **Gene** | **Name** | **Functions** | **References** |
| --- | --- | --- | --- |
| **Eid2** | E1A-like inhibitor of differentiation 2 | Inhibitor of gene expression in the muscle due to its capacity to block the histone acetyltransferase activity of the transcriptional co-activator p300 or to bind histone deacetylases. | (Ji et al., 2003) |
| **SOCS-2/6** | Suppressor of cytokine signaling 2 and 6 | Key controllers of the JAK/STAT signaling pathway.  SOCS2 prevents IL-2-induced STAT5 activation and development of Th2 cells.  SOCS2 contributes to the maintenance of Foxp3 expression in induced Tregs. | (Alexander et al., 2004)  (Knosp et al., 2011)  (Knosp et al., 2013) |
| **Cdkn2c** | Cyclin-dependent kinase inhibitor 2C | Inhibitor of cell cycle initiation and progression, modulation of TCR-mediated T cell proliferation. | (Kovalev et al., 2001) |
| **Tnfsf9** | Tumor necrosis factor superfamily, member 9 (CD137 ligand) | Ligand of CD137 (4-1BB), expressed by activated T cells, costimulatory role.  Inhibition of T cell proliferation and induction of apoptosis, induction of CD4^+^ T cell anergy. | (Nam et al., 2005; Shuford et al., 1997)  (Kwajah et al., 2011; Polte et al., 2007) |
| **Eomes** | Eomesodermin | Transcription factor inducing the expression of IFN-γ.  Cytotoxic CD8^+^ T cell differentiation and memory formation. | (Intlekofer et al., 2005; Pearce et al., 2003; Szabo et al., 2002) |
| **Map2k6** | Mitogen-activated protein kinase 6 | CD8^+^ T cell death | (Merritt et al., 2000) |
| **Mapk9** | c-Jun NH(2)-terminal kinase 2 (JNK2) | Negative regulator of cell proliferation | (Sabapathy et al., 2004) |
| **Pdcd7** | Programmed cell death 7 | Homologue ES18, positive regulation of T cell apoptotis | (Park et al., 1999) |
| **Cd7** | Galectin-1 receptor | Galectin-1 mediated apoptosis of activated T cells | (Koh et al., 2008; Stillwell et al., 2001) |
| **Id2** | Inhibitor of DNA binding 2 | Promotion of CD8^+^ T cell survival and repression of transition to memory state  Expression induced by the transcription factor Egr2 which also contributes to control T cell anergy. | (Cannarile et al., 2006; Masson et al., 2013)  (Kim et al., 2012; Safford et al., 2005) |
| **Hopx** | Homeodomain-only protein | Master regulator of the anergic state of induced Tregs.  Inhibitor of the AP-1 complex. | (Hawiger et al., 2010) |
| **Dap3** | Death-associated protein 3 | Ribosomal protein, mitochondrial apoptosis mediator | (Miyazaki et al., 2001) |
| **Stim1** | Stromal interaction molecule 1 | Endoplasmic reticulum Ca2^+^ sensor, upregulated during T cell activation, recruited to the immunological synapse. | (Lioudyno et al., 2008; Zhang et al., 2005) |
| **Tgfbr3** | Transforming growth factor-β receptor 3/Betaglycan | Signaling co-receptor for TGFβ superfamily ligands: TGFβ1-3, inhibin, Bone morphogenetic proteins and fibroblast growth factor.  Inhibition of cellular migration through an β-arrestin2-dependent mechanism. | (Mythreye et al., 2009; Mythreye et al., 2009) |
| **Fcgr2b** | Fc gamma receptor 2b | Inhibitory Fc receptor, control of plasma cell persistence and apoptosis, inhibition of effector T cell responses. | (Nimmerjahn et al., 2008; Xiang et al., 2007) |
| **Btla** | B and T lymphocyte attenuator | Negative costimulatory molecule, highly expressed on anergic CD4^+^ T cells | (Hurchla et al., 2005) |

**Supplemental references**

Alexander WS and Hilton DJ. 2004. The role of suppressors of cytokine signaling (SOCS) proteins in regulation of the immune response. *Annu Rev Immunol* **22**:503-29.

Cannarile MA, Lind NA, Rivera R, Sheridan AD, Camfield KA, Wu BB, Cheung KP, Ding Z and Goldrath AW. 2006. Transcriptional regulator Id2 mediates CD8+ T cell immunity. *Nat Immunol* **7**:1317-25.

Hawiger D, Wan YY, Eynon EE and Flavell RA. 2010. The transcription cofactor Hopx is required for regulatory T cell function in dendritic cell-mediated peripheral T cell unresponsiveness. *Nat Immunol* **11**:962-8.

Hurchla MA, Sedy JR, Gavrieli M, Drake CG, Murphy TL and Murphy KM. 2005. B and T lymphocyte attenuator exhibits structural and expression polymorphisms and is highly Induced in anergic CD4+ T cells. *J Immunol* **174**:3377-85.

Intlekofer AM, Takemoto N, Wherry EJ, Longworth SA, Northrup JT, Palanivel VR, Mullen AC, Gasink CR, Kaech SM, Miller JD, Gapin L, Ryan K, Russ AP, Lindsten T, Orange JS, Goldrath AW, Ahmed R and Reiner SL. 2005. Effector and memory CD8+ T cell fate coupled by T-bet and eomesodermin. *Nat Immunol* **6**:1236-44.

Ji A, Dao D, Chen J and MacLellan WR. 2003. EID-2, a novel member of the EID family of p300-binding proteins inhibits transactivation by MyoD. *Gene* **318**:35-43.

Kim HJ, Hong JM, Yoon KA, Kim N, Cho DW, Choi JY, Lee IK and Kim SY. 2012. Early growth response 2 negatively modulates osteoclast differentiation through upregulation of Id helix-loop-helix proteins. *Bone* **51**:643-50.

Knosp CA, Carroll HP, Elliott J, Saunders SP, Nel HJ, Amu S, Pratt JC, Spence S, Doran E, Cooke N, Jackson R, Swift J, Fitzgerald DC, Heaney LG, Fallon PG, Kissenpfennig A and Johnston JA. 2011. SOCS2 regulates T helper type 2 differentiation and the generation of type 2 allergic responses. *J Exp Med* **208**:1523-31.

Knosp CA, Schiering C, Spence S, Carroll HP, Nel HJ, Osbourn M, Jackson R, Lyubomska O, Malissen B, Ingram R, Fitzgerald DC, Powrie F, Fallon PG, Johnston JA and Kissenpfennig A. 2013. Regulation of Foxp3+ inducible regulatory T cell stability by SOCS2. *J Immunol* **190**:3235-45.

Koh HS, Lee C, Lee KS, Ham CS, Seong RH, Kim SS and Jeon SH. 2008. CD7 expression and galectin-1-induced apoptosis of immature thymocytes are directly regulated by NF-kappaB upon T-cell activation. *Biochem Biophys Res Commun* **370**:149-53.

Kovalev GI, Franklin DS, Coffield VM, Xiong Y and Su L. 2001. An important role of CDK inhibitor p18(INK4c) in modulating antigen receptor-mediated T cell proliferation. *J Immunol* **167**:3285-92.

Kwajah MMS, Mustafa N, Holme AL, Pervaiz S and Schwarz H. 2011. Biphasic activity of CD137 ligand-stimulated monocytes on T cell apoptosis and proliferation. *J Leukoc Biol* **89**:707-20.

Lioudyno MI, Kozak JA, Penna A, Safrina O, Zhang SL, Sen D, Roos J, Stauderman KA and Cahalan MD. 2008. Orai1 and STIM1 move to the immunological synapse and are up-regulated during T cell activation. *Proc Natl Acad Sci U S A* **105**:2011-6.

Masson F, Minnich M, Olshansky M, Bilic I, Mount AM, Kallies A, Speed TP, Busslinger M, Nutt SL and Belz GT. 2013. Id2-mediated inhibition of E2A represses memory CD8+ T cell differentiation. *J Immunol* **190**:4585-94.

Merritt C, Enslen H, Diehl N, Conze D, Davis RJ and Rincon M. 2000. Activation of p38 mitogen-activated protein kinase in vivo selectively induces apoptosis of CD8(+) but not CD4(+) T cells. *Mol Cell Biol* **20**:936-46.

Miyazaki T and Reed JC. 2001. A GTP-binding adapter protein couples TRAIL receptors to apoptosis-inducing proteins. *Nat Immunol* **2**:493-500.

Mythreye K and Blobe GC. 2009. Proteoglycan signaling co-receptors: roles in cell adhesion, migration and invasion. *Cell Signal* **21**:1548-58.

Mythreye K and Blobe GC. 2009. The type III TGF-beta receptor regulates epithelial and cancer cell migration through beta-arrestin2-mediated activation of Cdc42. *Proc Natl Acad Sci U S A* **106**:8221-6.

Nam KO, Kang H, Shin SM, Cho KH, Kwon B, Kwon BS, Kim SJ and Lee HW. 2005. Cross-linking of 4-1BB activates TCR-signaling pathways in CD8+ T lymphocytes. *J Immunol* **174**:1898-905.

Nimmerjahn F and Ravetch JV. 2008. Fcgamma receptors as regulators of immune responses. *Nat Rev Immunol* **8**:34-47.

Park EJ, Kim JH, Seong RH, Kim CG, Park SD and Hong SH. 1999. Characterization of a novel mouse cDNA, ES18, involved in apoptotic cell death of T-cells. *Nucleic Acids Res* **27**:1524-30.

Pearce EL, Mullen AC, Martins GA, Krawczyk CM, Hutchins AS, Zediak VP, Banica M, DiCioccio CB, Gross DA, Mao C, Shen H, Cereb N, Yang SY, Lindsten T, Rossant J, Hunter CA and Reiner SL. 2003. Control of effector CD8+ T cell function by the transcription factor Eomesodermin. *Science* **302**:1041-43.

Polte T, Jagemann A, Foell J, Mittler RS and Hansen G. 2007. CD137 ligand prevents the development of T-helper type 2 cell-mediated allergic asthma by interferon-gamma-producing CD8+ T cells. *Clin Exp Allergy* **37**:1374-85.

Sabapathy K and Wagner EF. 2004. JNK2: a negative regulator of cellular proliferation. *Cell Cycle* **3**:1520-3.

Safford M, Collins S, Lutz MA, Allen A, Huang CT, Kowalski J, Blackford A, Horton MR, Drake C, Schwartz RH and Powell JD. 2005. Egr-2 and Egr-3 are negative regulators of T cell activation. *Nat Immunol* **6**:472-80.

Shuford WW, Klussman K, Tritchler DD, Loo DT, Chalupny J, Siadak AW, Brown TJ, Emswiler J, Raecho H, Larsen CP, Pearson TC, Ledbetter JA, Aruffo A and Mittler RS. 1997. 4-1BB costimulatory signals preferentially induce CD8+ T cell proliferation and lead to the amplification in vivo of cytotoxic T cell responses. *J Exp Med* **186**:47-55.

Stillwell R and Bierer BE. 2001. T cell signal transduction and the role of CD7 in costimulation. *Immunol Res* **24**:31-52.

Szabo SJ, Sullivan BM, Stemmann C, Satoskar AR, Sleckman BP and Glimcher LH. 2002. Distinct effects of T-bet in TH1 lineage commitment and IFN-gamma production in CD4 and CD8 T cells. *Science* **295**:338-42.

Xiang Z, Cutler AJ, Brownlie RJ, Fairfax K, Lawlor KE, Severinson E, Walker EU, Manz RA, Tarlinton DM and Smith KG. 2007. FcgammaRIIb controls bone marrow plasma cell persistence and apoptosis. *Nat Immunol* **8**:419-29.

Zhang SL, Yu Y, Roos J, Kozak JA, Deerinck TJ, Ellisman MH, Stauderman KA and Cahalan MD. 2005. STIM1 is a Ca2+ sensor that activates CRAC channels and migrates from the Ca2+ store to the plasma membrane. *Nature* **437**:902-5.
